# Efficient Architecture for Spike Sorting in Reconfigurable Hardware

**DOI:** 10.3390/s131114860

**Published:** 2013-11-01

**Authors:** Wen-Jyi Hwang, Wei-Hao Lee, Shiow-Jyu Lin, Sheng-Ying Lai

**Affiliations:** 1 Department of Computer Science and Information Engineering, National Taiwan Normal University, Taipei 116, Taiwan; E-Mails: gdset@hotmail.com (W.-H.L.); 699470125@ntnu.edu.tw (S.-Y.L.); 2 Department of Electronic Engineering, National Ilan University, Yilan 260, Taiwan; E-Mail: sjlin@niu.edu.tw

**Keywords:** spike sorting, reconfigurable computing, system-on-chip, generalized Hebbian algorithm, fuzzy C-means, FPGA

## Abstract

This paper presents a novel hardware architecture for fast spike sorting. The architecture is able to perform both the feature extraction and clustering in hardware. The generalized Hebbian algorithm (GHA) and fuzzy C-means (FCM) algorithm are used for feature extraction and clustering, respectively. The employment of GHA allows efficient computation of principal components for subsequent clustering operations. The FCM is able to achieve near optimal clustering for spike sorting. Its performance is insensitive to the selection of initial cluster centers. The hardware implementations of GHA and FCM feature low area costs and high throughput. In the GHA architecture, the computation of different weight vectors share the same circuit for lowering the area costs. Moreover, in the FCM hardware implementation, the usual iterative operations for updating the membership matrix and cluster centroid are merged into one single updating process to evade the large storage requirement. To show the effectiveness of the circuit, the proposed architecture is physically implemented by field programmable gate array (FPGA). It is embedded in a System-on-Chip (SOC) platform for performance measurement. Experimental results show that the proposed architecture is an efficient spike sorting design for attaining high classification correct rate and high speed computation.

## 1. Introduction

A spike train is a sequence of action potentials generated by a neuron. Extracellular neural recording often results in a mixture of these trains from neurons near the recording electrodes. Spike sorting is the process of segregating the spike trains of individual neurons from this mixture. Spike sorting is a difficult task, due to the presence of background noise and the interferences among neurons in a local area. A typical spike sorting algorithm involves computationally demanding operations, such as feature extraction and clustering [1]. For applications, such as brain machine interface (BMI) [2], involving the control of artificial limb movements, spike sorting systems need to have the ability to process raw spike trains in real time, because the typical delay between neural activity and human limb movement is only several hundred milliseconds [3]. One way to expedite the spike sorting computation is to implement the algorithms in hardware.

A common approach for hardware design is based on application-specific integrated circuits (ASICs). A major drawback of ASICs is the lack of flexibility for changes. With the wide range of spike sorting algorithms that already exist and the continual design and improvement of algorithms, the ability to easily change a spike sorting system for new algorithms is usually desired. However, the modifications in ASIC are very difficult, especially when chips are implanted in the brain. The field programmable gate array (FPGA) [4] is an effective alternative to ASIC for hardware implementation with lower NREcosts. Moreover, the circuits in an FPGA are reconfigurable, thereby providing higher flexibility to a spike sorting architecture for future extensions.

The goal of this paper is to present an FPGA-based spike sorting hardware architecture for real-time computation. The architecture is able to perform both feature extraction and clustering in hardware, which are the most computationally demanding tasks for spike sorting. The feature extraction is based on the generalized Hebbian algorithm (GHA) [5,6], which is an incremental principal component analysis (PCA) algorithm capable of extracting PCA features without the need of covariance matrix of input data. Therefore, the GHA is more suitable for hardware implementation, because there is no computation and storage overhead for processing covariance matrix. The resulting features are then clustered using the fuzzy C-means (FCM) algorithm [7,8]. As compared with other unsupervised clustering algorithms, such as K-means, the FCM algorithm has the advantage that its performance is less sensitive to the selection of initial centers. Therefore, it is less likely for the FCM-based feature clustering to fall into a poor local optimum.

A challenging issue of combining both GHA and FCM circuits in one chip is the high area costs. To reduce the hardware resource utilization, a spike is separated into a number of smaller blocks in the GHA circuit. Different blocks share the same circuit for PCA feature extraction. In addition, the FCM circuit pre-computes the common factors of different membership coefficients, so that the number of adders and multipliers for membership coefficients computation can be reduced. The requirement for storing entire membership coefficients matrix for center computation can also be evaded by adopting an incremental center computation scheme in the FCM circuit.

To physically evaluate the proposed architecture, a spike sorting system on an FPGA-based System-on-Chip (SOC) platform is implemented, where the proposed spike sorting architecture is used as a hardware accelerator. The softcore processor in the SOC platform is adopted for coordinating different components in the SOC. It is also used to measure the computation time of the proposed architecture. Experimental results reveal that the proposed architecture is an effective alternative for real-time spike sorting with accurate feature extraction and clustering.

## 2. Related Works

A software implementation of PCA and FCM for automatic and online spike sorting is implemented in [9]. It uses a partial single value decomposition (PSVD) preprocessing technique for enhancing the robustness and speed of PCA. A spike sorting system based on SOC is proposed in [10]. The system is implemented on the Smartdust platform, in which PCA-based feature extraction and K-means-based clustering are realized by software. For these software approaches, real-time spike sorting operations may be difficult when the processor operates at low clock rates. A number of FPGA-based hardware architectures have been proposed to expedite the spike sorting. The architectures in [11,12] are able to perform online PCA/GHA feature extraction. The architectures for discrete wavelet transform (DWT)-based feature extraction are proposed in [13,14]. The circuit for the extraction of zero-crossing features (ZCF) of spike trains is presented in [15]. The architecture in [16] is capable of performing self-organizing map (SOM)-based clustering. A common drawback of these architectures is that they are not able to perform both feature extraction and clustering. Because all of these operations are required for spike sorting, hardware implementation of any single operation may not be able to provide sufficient throughput at the front end.

Studies in [17–19] have proposed GHA architectures for texture classification and face recognition. Although these architecture may be directly used for spike sorting, some architectures are not suited, because of their high area costs and/or long latency. The architecture presented in [17] provides high throughput for GHA training, since it processes all elements of input vectors concurrently. However, the area cost of the architecture grows linearly with the dimension of input vectors. On the contrary, only one element of input vectors is delivered to architecture proposed in [18] at a time. The architecture therefore has low area cost. Nevertheless, its latency grows linearly with the vector dimension. Hence, the architecture may not be suitable for the training of long spikes. The architecture in [19] separates input vectors into a number of smaller blocks. It then processes one block at a time. The architecture has both the advantages of low area costs and high computational speed [19].

In addition to GHA architectures, many FCM architectures [20–22] have been proposed for image processing. However, some of these architecture are difficult to be extended for spike sorting. The architecture in [20] is designed for clustering with only two classes. For spike sorting applications, the number of classes may be larger than two. The architecture in [21] allows all the membership coefficients associated with a training vector to be computed in parallel. The architecture has both high throughput and high area costs. The high hardware utilization increases the difficulty of integrating FCM with GHA on a single chip. An effective alternative to [20,21] is based on [22], which produces membership coefficients sequentially to reduce the area costs. In addition, the common factors of different membership coefficients are pre-computed by a shared circuit to further lower the hardware resource utilization. This pre-computation step is also able to accelerate the speed of membership coefficient computation.

## 3. Preliminaries

This section briefly review some basic facts of GHA and FCM algorithms and their applications to spike sorting.

### 3.1. GHA

Let:

(1) $$\text{x}(n)={\left[x_{1}(n),\ldots ,x_{m}(n)\right]}^{T},n=1,\ldots ,t$$

(2) $$\text{y}(n)={\left[y_{1}(n),\ldots ,y_{p}(n)\right]}^{T},n=1,\ldots ,t$$

be the *n*-th input and output vectors to the GHA, respectively. In addition, *m*, *p* and *t* are the vector dimension, the number of principal components (PCs) and the number of input and output vectors for the GHA, respectively. The output vector, **y**(*n*), is related to the input vector, **x**(*n*), by:

(3) $$y_{j}(n)=\sum_{i=1}^{m}w_{ji}(n)x_{i}(n)$$

where the *w_ji_*(*n*) stands for the weight from the *i*-th synapse to the *j*-th neuron at iteration *n*.

Let:

(4) $${\text{w}}_{j}(n)={\left[w_{j1}(n),\ldots ,w_{jm}(n)\right]}^{T},j=1,\ldots ,p$$

be the *j*-th synaptic weight vector. Each synaptic weight vector, **w***_j_*(*n*), is adapted by the Hebbian learning rule:

(5) $$w_{ji}(n+1)=w_{ji}(n)+\eta [y_{j}(n)x_{i}(n)-y_{j}(n)\sum_{k=1}^{j}w_{ki}(n)y_{k}(n)]$$

where *η* denotes the learning rate. After a great deal of iterative computation and adaptation, **w***_j_* (*n*) will asymptotically approach the eigenvector associated with the *j*-th eigenvalue, λ*_j_*, of the covariance matrix of input vectors, where λ_1_ > λ_2_ >⋯> λ*_p_*. To reduce the complexity of computing implementation, Equation (5) can be rewritten as:

(6) $$w_{ij}(n+1)=w_{ij}(n)+\eta y_{j}(n)[x_{i}(n)-\sum_{k=1}^{j}w_{ki}(n)y_{k}(n)]$$

A more detailed discussion of GHA can be found in [5,6].

### 3.2. FCM

Let *F* = {**f**_1_, …,**f***_t_*} be a set of training vectors for clustering, where *t* is the number of training vectors. The FCM computes v*_i_*, *i* = 1, …, *c*, to separate *F* into *c* clusters. The **v***_i_* is the center of cluster *i*. The FCM involves minimization of the following cost function:

(7) $$J=\sum_{i=1}^{c}\sum_{n=1}^{t}u_{i,n}^{2}{\left\|{\text{f}}_{n}-{\text{v}}_{i}\right\|}^{2}$$

where *u_i,n_* is the membership of **x***_k_* in class *i*. The cost function, *J*, is minimized by a two-step iteration in the FCM. In the first step, the centers, **v**_1_,…, **v***_c_*, are fixed, and the optimal membership matrix, {*u_i,n_*, *i* = 1,…, *c*, *n* = 1,…, *t*}, is computed by:

(8) $$u_{i,n}={\left(\sum_{j=1}^{c}{\left(\left\|{\text{f}}_{n}-{\text{v}}_{i}\right\|/\left\|{\text{f}}_{n}-{\text{v}}_{j}\right\|\right)}^{2}\right)}^{-1}$$

After the first step, the membership matrix is then fixed, and the new center, **v***_i_*, is obtained by:

(9) $${\text{v}}_{i}=(\sum_{n=1}^{t}u_{i,n}^{2}{\text{f}}_{n})/(\sum_{n=1}^{t}u_{i,n}^{2})$$

The iteration continues until the convergence of *J*.

### 3.3. Applications of GHA and FCM to Spike Sorting

The GHA and FCM can be used for feature extraction and clustering of spikes, respectively. To use GHA for feature extraction, the x(*n*) in Equation (2) is the *n*-th spike in the spike train. Therefore, the vector dimension, *m*, is the number of samples in a spike. Let:

(10) $${\text{w}}_{j}={\left[w_{j1},\ldots ,w_{jm}\right]}^{T},j=1,\ldots ,p$$

be the synaptic weight vectors of the GHA after the training process has completed. Based on **w***_j_*, *j* = 1, …, *p*, the GHA feature vector extracted from training vector, **x**(*n*) (denoted by **f***_n_*), is computed by:

(11) $${\text{f}}_{n}={\left[f_{n,1},\ldots ,f_{n,p}\right]}^{T}$$

where:

(12) $$f_{n,j}=\sum_{i=1}^{m}w_{ji}x_{i}(n)$$

is the *j*-th element of **f***_n_*. The set of feature vectors *F* = {**f**_1_, …, **f***_t_*} are then used as the training set for FCM. After the FCM training shown in Section 3.2 is completed, the resulting cluster centers, **v**_1_, …, **v***_c_*, are then used to cluster the spikes. The clustering is based on the following simple rule that spike **x**(*n*) is assigned to cluster *i* if:

(13) $$i=\arg\underset{1\leq j\leq c}{\min}d({\text{f}}_{n},{\text{v}}_{j})$$

where *d*(**f***_n_*, **v***_j_*) is the squared distance between **f***_n_* and **v***_j_*.

## 4. The Proposed Architecture

**Figure 1.**
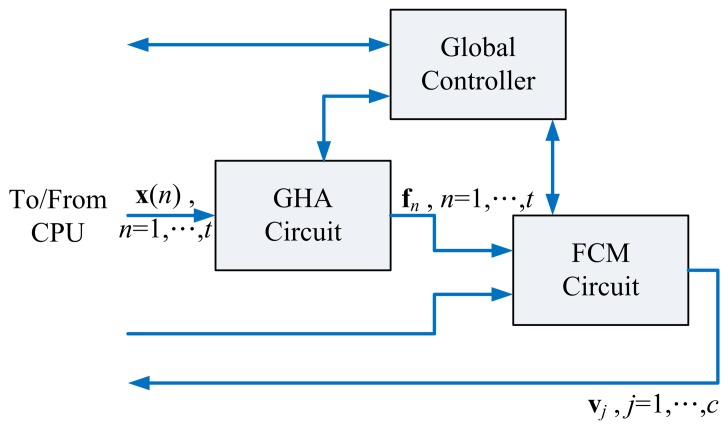
The proposed spike sorting architecture.

Figure 1 shows the proposed architecture for spike sorting, which contains the GHA unit, FCM unit and global controller. Based on spikes **x**(*n*), *n* = 1, …, *t*, the GHA unit is used for computing feature vectors **f***_n_*, *n* = 1, …, *t*. The FCM unit then computes centers **v***_j_*, *j* = 1, …*c*, for clustering. The global controller coordinates all the components in the architecture.

### 4.1. GHA Unit

The architecture of the GHA unit is an extension of the architecture presented in [19]. The GHA unit consists of three functional units: the memory unit, the synaptic weight updating (SWU) unit and the principal components computing (PCC) unit.

### 4.2. SWU Unit of GHA

The design of the SWU unit is based on Equation (6). Although the direct implementation of Equation (6) is possible, it will consume large hardware resources [19]. One way to reduce the resource consumption is by observing that Equation (6) can be rewritten as:

(14) $$w_{ij}(n+1)=w_{ij}(n)+\eta y_{j}(n)z_{ji}(n)$$

where:

(15) $$z_{ij}(n)=x_{i}(n)-\sum_{k=1}^{j}w_{ki}(n)y_{k}(n),j=1,\ldots ,p$$

and **z***_j_*(*n*) = [*z_j_*_1_(*n*), …, *z_jm_*(*n*)]*^T^*. The *z_ji_*(*n*) can be obtained from *z*_(_*_j_*_−1)_*_i_*(*n*) by:

(16) $$z_{ji}(n)=z_{(j-1)i}(n)-w_{ji}(n)y_{j}(n),j=2,\ldots ,p$$

When *j* = 1, from Equations (15) and (16), it follows that:

(17) $$z_{0i}(n)=x_{i}(n)$$

**Figure 2.**
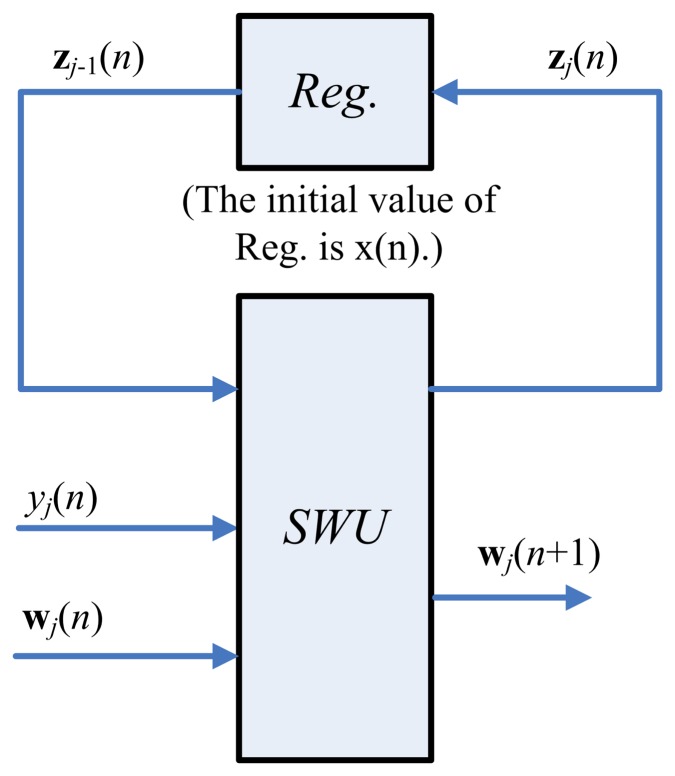
The hardware implementation of Equations (14) and (16).

Therefore, the hardware implementation of Equations (14) and (16) is equivalent to that of Equation (6). Figure 2 depicts the hardware implementation of Equations (14) and (16). As shown in the figure, the SWU unit produces one synaptic weight vector at a time. The computation of **w***_j_*(*n*+1), the *j*-th weight vector at the iteration *n*+1, requires the **z***_j_*_−1_(*n*), **y**(*n*) and **w***_j_*(*n*) as inputs. In addition to **w***_j_*(*n*+1), the SWU unit also produces **z***_j_*(*n*), which will then be used for the computation of **w***_j_*_+1_(*n* + 1). Hardware resource consumption can then be effectively reduced.

**Figure 3.**
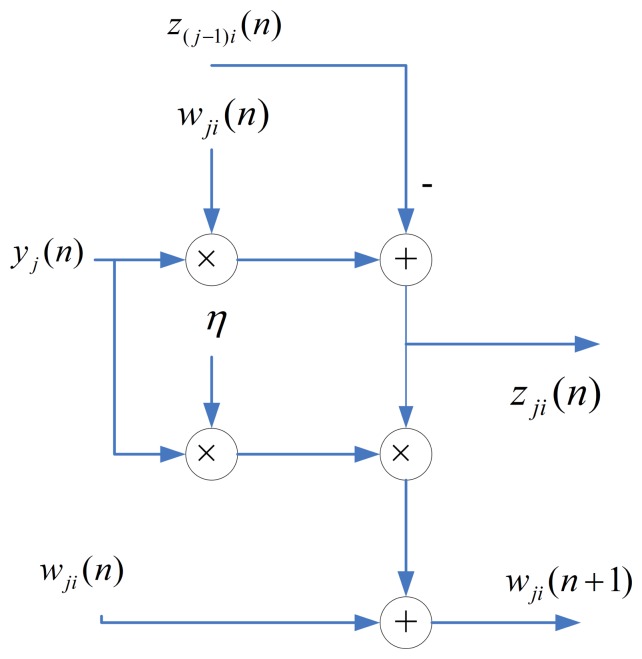
The architecture of each module in the synaptic weight updating (SWU) unit.

One way to implement the SWU unit is to produce **w***_j_*(*n* + 1) and **z***_j_*(*n*) in one shot. However, *m* identical modules, individually shown in Figure 3, may be required, because the dimension of vectors is *m*. The area costs of the SWU unit then grow linearly with *m*. To further reduce the area costs, each of the output vectors, **w***_j_*(*n*+1) and **z***_j_*(*n*), is separated into *b* blocks, where each block contains *q* elements. The SWU unit only computes one block of **w***_j_*(*n*+1) and **z***_j_*(*n*) at a time. Therefore, it will take *b* clock cycles to produce complete **w***_j_*(*n* + 1) and **z***_j_*(*n*).

### 4.3. PCC Unit of GHA

The PCC operations are based on Equation (3). Therefore, the PCC unit of the proposed architecture contains adders and multipliers. Because the number of multipliers grows with the vector dimension, *m*, the direct implementation using Equation (3) may consume large hardware resources when *m* becomes large. Similar to the SWU unit, the block-based computation is used for reducing the area costs. In fact, Equation (3) can be rewritten as:

(18) $$y_{j}(n)=\sum_{k=1}^{b}\sum_{i=1}^{q}w_{j,(k-1)q+i}(n)x_{(k-1)q+i}(n)$$

The implementation of Equation (18) needs only *q* multipliers, a *q*-input adder and an accumulator.

### 4.4. Memory Unit of GHA

The memory unit contains four buffers: Buffers A, B, C and D. Buffer A fetches and stores spike x(*n*) from the main memory. Buffer B contains **z***_j_*(*n*) for the computation in PCC and SWU units. Buffer C consists of the synaptic weight vectors, **w***_j_*(*n*). The feature vectors, **f**_1_, …, **f***_t_*, are stored in Buffer D. The Buffers A, B and C are shift registers. Buffer D is a two-port RAMfor the subsequent access by the FCM unit.

### 4.5. Operations of the GHA Unit

**Figure 4.**
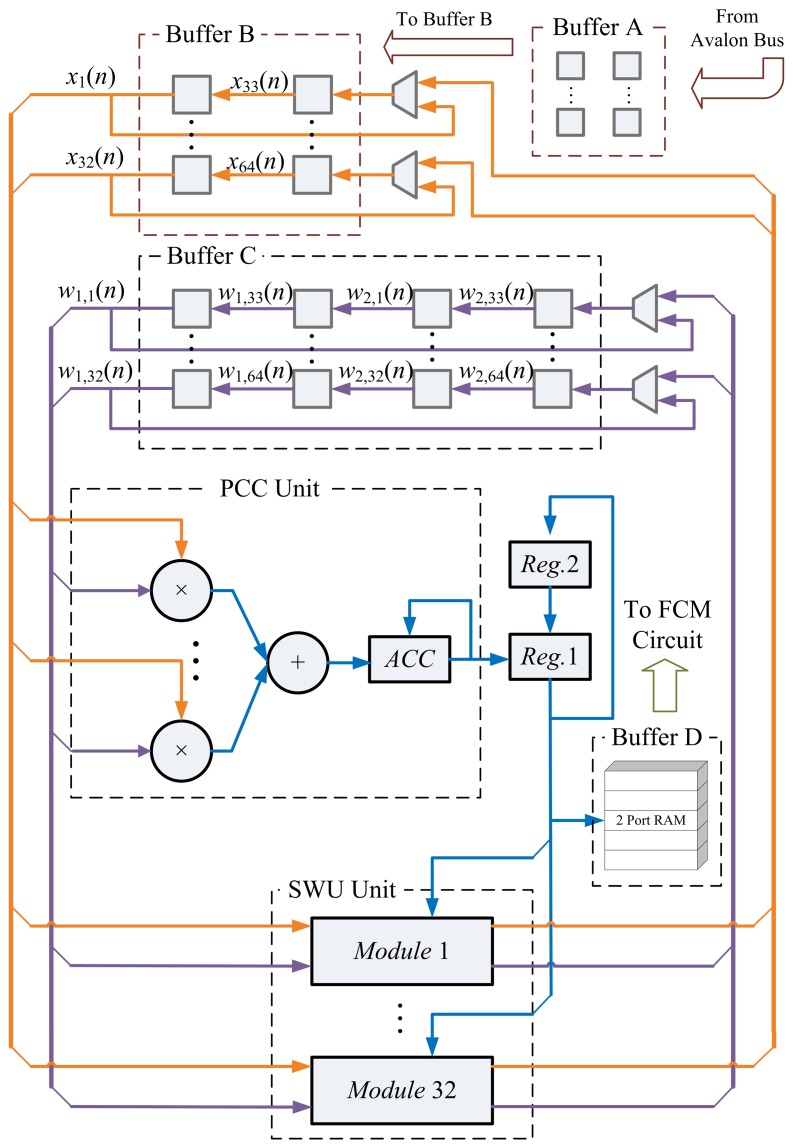
The generalized Hebbian algorithm (GHA) circuit for *m* = 64, *p* = 2, *b* = 2 and *q* = 32.

**Figure 5.**
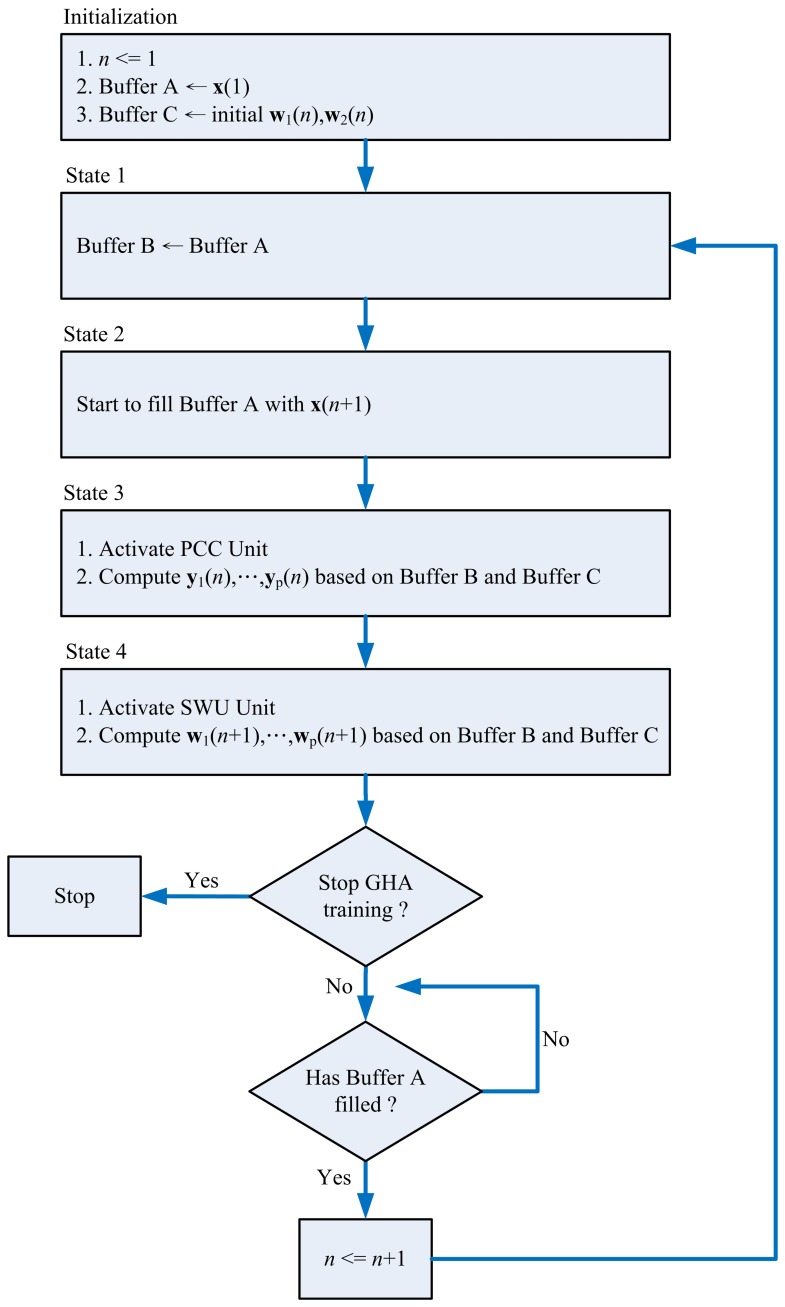
The training operations of the GHA circuit.

In typical spike sorting implementations [10,23], a spike may contain 64 samples. In addition, two PCs may suffice for feature extraction [1]. Therefore, without loss of generality, the GHA unit for *m* = 64 (*i.e.*, the vector dimension is 64) and *p* = 2 (*i.e.*, number of PCs is two) is considered in this subsection. In the GHA unit, each vector is separated into two blocks. Moreover, the dimension of each block is 32. Therefore, we set *b* = 2 and *q* = 32 for the circuit implementation. Figure 4 shows the resulting GHA circuit for *m* = 64, *p* = 2, *b* = 2 and *q* = 32. The operations of the GHA circuit can be separated into four states, as revealed in Figure 5. The most important operations of the GHA circuit are the PCC operations in State 3 and SWU operations in State 4. These two operations are further elaborated below.

**Figure 6.**
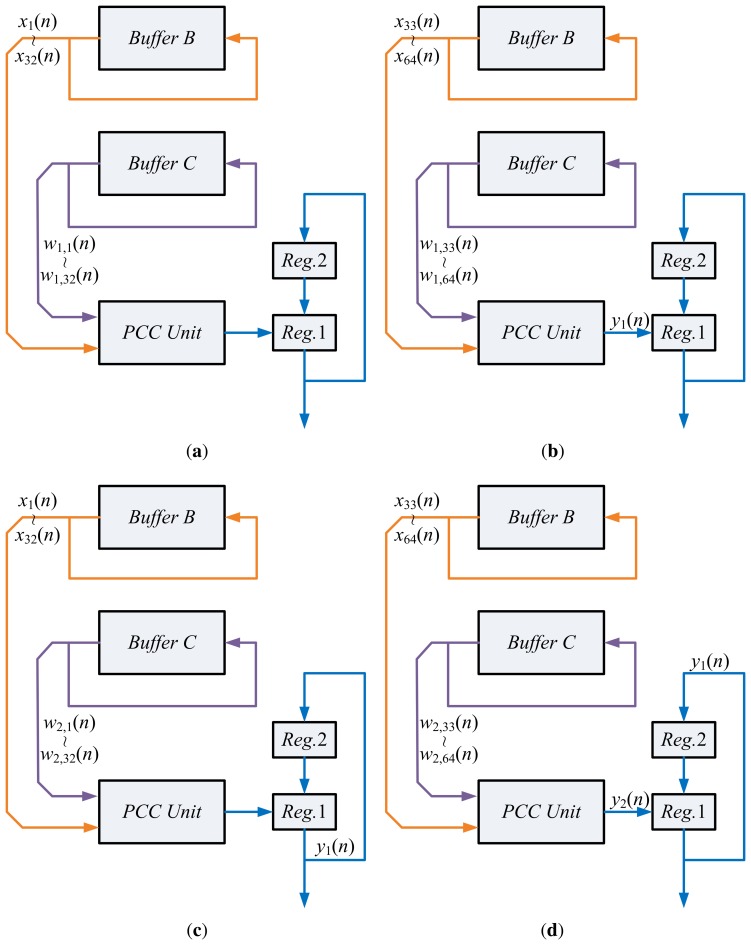
Operations of principal components computing (PCC) unit in State 3 of the GHA circuit: (**a**) first step of computing *y*_1_(*n*); (**b**) second step of computing *y*_1_(*n*); (**c**) first step of computing *y*_2_(*n*); (**d**) second step of computing *y*_2_(*n*).

Assume the input vector, **x**(*n*), is available in Buffer B. In addition, the *current* synaptic weight vectors, **w**_1_(*n*),**w**_2_(*n*), are stored in the Buffer C. Based on **x**(*n*) and **w**_1_(*n*),**w**_2_(*n*) the PCC unit produces output vector *y*_1_(*n*), *y*_2_(*n*). Figure 6 reveals the operations of the PCC unit for computing *y*_1_(*n*), *y*_2_(*n*). The computation of *y_j_*(*n*) is separated into two steps. The first step finds $\sum_{i=1}^{32}w_{j,i}(n)x_{i}(n)$. The second step computes $\sum_{i=33}^{64}w_{j,i}(n)x_{i}(n)$ and, then, accumulates the result with that of the previous step to find *y_j_*(*n*). These two steps share the same circuit. It can also be observed from Figure 6 that the data obtained from Buffers B and C for PCC operations are also rotated back to Buffers B and C for subsequent operations in the SWU unit.

**Figure 7.**
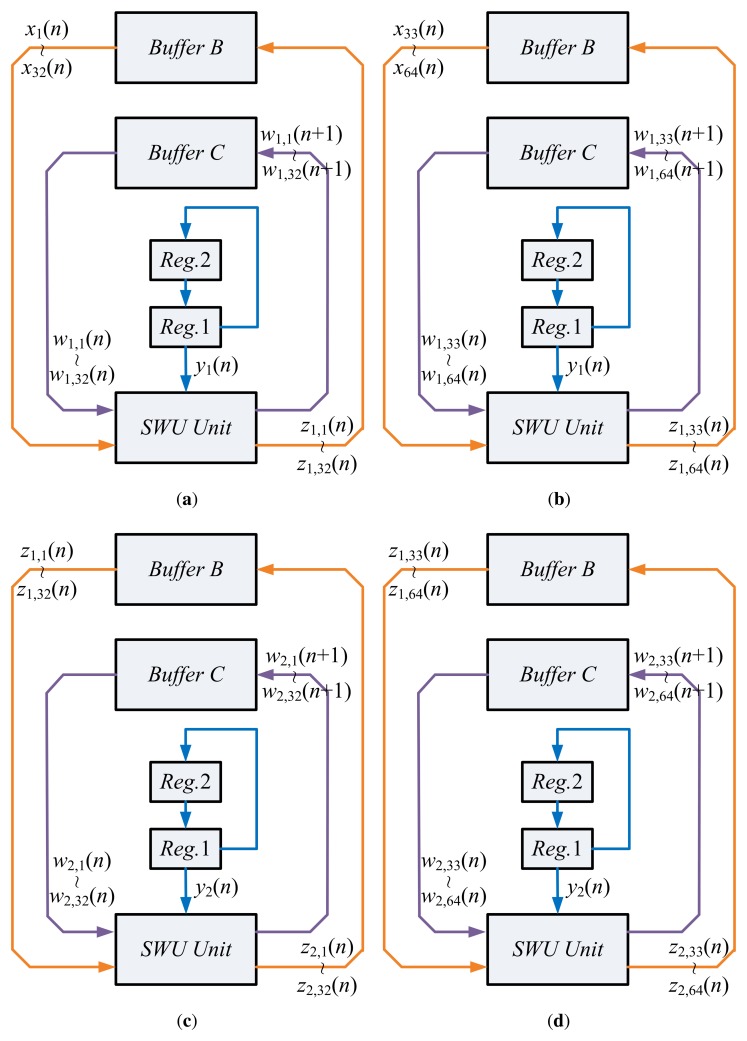
Operations of SWU unit in State 4 of the GHA circuit: (**a**) first step of computing **w**_1_(*n* + 1); (**b**) second step of computing **w**_1_(*n* + 1); (**c**) first step of computing **w**_2_(*n* + 1); (**d**) second step of computing **w**_2_(*n* + 1).

Upon the completion of PCC operations, the SWU unit will be activated in State 4. Figure 7 shows the operations of the SWU unit. Using **x**(*n*), *y_j_*(*n*) and **w***_j_*(*n*), *j* = 1, 2, the SWU unit computes the new synaptic weight vectors, **w***_j_*(*n* + 1), *j* = 1, 2, which are then stored back to Buffer C for subsequent training. Similar to the computation of *y_j_*(*n*), the computation of **w***_j_*(*n* + 1) consists of two steps. The first step computes the first half of **w***_j_*(*n*+1) (*i.e.*, *w_j_*_,1_(*n*+1),…,*w_j_*_,32_(*n*+1)). The second step calculates the second half. These two steps also share the same circuit. Moreover, the computation of **w**_1_(*n* + 1) also produces **z**_1_(*n*), which is stored back to Buffer B. As revealed in Figure 7c,d, **z**_1_(*n*) is then retrieved from Buffer B for the computation of **w**_2_(*n* + 1).

After the training process of the GHA circuit is completed, Buffer C contains the synaptic weight vectors, **w**_1_ and **w**_2_. Based on the synaptic weight vectors, the feature extraction operations can then proceed, which are the same as those shown in Figure 6 for PCC operations. The computation results are stored in Buffer D as feature vectors **f***_n_*, *n* = 1, …, *t*, for the subsequent FCM clustering operations.

### 4.6. FCM Unit

The architecture of the FCM unit contains six sub-units: the pre-computation unit, the membership coefficients updating unit, centroid updating unit, cost function computation unit, FCM memory unit and control unit. The operations of each sub-unit are stated below.

### 4.7. Pre-Computation Unit of FCM

The pre-computation unit is used for reducing the computational complexity of the membership coefficients calculation. Observe that *u_i,n_* in Equation (8) can be rewritten as:

(19) $$u_{i,n}={\left\|{\text{f}}_{n}-{\text{v}}_{i}\right\|}^{-2}P_{n}^{-1}$$

where:

(20) $$P_{n}=\sum_{j=1}^{c}\left(1/{\left\|{\text{f}}_{n}-{\text{v}}_{j}\right\|}^{2}\right)$$

Given **f***_n_* and centers **v**_1_, …, **v***_c_*, membership coefficients *u*_1_,*_n_*, …, *u_c,n_* have the same *P_n_*. Therefore, the complexity for computing membership coefficients can be reduced by calculating *P_n_* in the pre-computation unit. Based on Equation (20), the architecture for computing *P_n_* can be divided into two stages. The first stage evaluates ‖**f***_n_* − *v_j_*‖^2^. The second stage first finds the inverse of ‖**f***_n_* − *v_j_*‖^2^ and, then, accumulate this value with $\sum_{j=1}^{i=1}1/{\left\|{\text{f}}_{n}-{\text{v}}_{j}\right\|}^{2}.$

### 4.8. Membership Updating Unit of FCM

Based on Equation (19), the membership updating unit uses the computation results of the precomputation unit for calculating the membership coefficients. Given **f***_n_*, the membership coefficients computation unit computes $u_{i,n}^{2}$ for *i* = 1, …, *c*, one at a time. The circuit can be separated to two stages. The first stage is used for computing ‖**f***_n_* − **v***_i_*‖^2^*P_n_*. The membership coefficient, $u_{i,n}^{2}$, is then obtained by a cascade of inverse and square operations.

### 4.9. Center Updating Unit of FCM

The center updating unit incrementally computes the center of each cluster. The major advantage for the incremental computation is that it is not necessary to store the entire membership coefficients matrix for the center computation. Define the incremental center for the *i*-th cluster up to **f***_n_* as:

(21) $${\text{v}}_{i}(n)=(\sum_{k=1}^{n}{\text{f}}_{k}u_{i.k}^{2})/(\sum_{k=1}^{n}u_{i,k}^{n})$$

when *n* = *t*, **v***_i_*(*n*) then is identical to the actual center **v***_i_* given in Equation (9).

The center update unit operates in accordance with Equation (21). It contains a multiplier, a cell array and a divider. There are two groups of cells in the cell array. The cell *i* in the first group contains the accumulated sum $\sum_{k=1}^{n-1}{\text{f}}_{k}u_{i,k}^{2}$. Moreover, the cell *i* in the second group contains the accumulated sum $\sum_{k=1}^{n-1}u_{i,k}^{2}$. Therefore, each cell in the array is actually an accumulator. The outputs of the array are used for computing **v***_i_*(*n*) using a divider.

### 4.10. Cost Function Computation Unit of FCM

Similar to the centroid updating unit, the cost function unit incrementally computes the cost function, *J*. Define the incremental cost function, *J*(*i, k*), as:

(22) $$J(i,n)=\sum_{k=1}^{n}\sum_{j=1}^{i}u_{j,z}^{2}{\left\|{\text{f}}_{k}-{\text{v}}_{j}\right\|}^{2}$$

The circuit receives $u_{i,n}^{2}$ and ‖**f***_n_* − **v***_i_*‖^2^ from the membership coefficient updating unit. The product $u_{i,n}^{2}{\left\|{\text{f}}_{n}-{\text{v}}_{i}\right\|}^{2}$ is then accumulated for computing *J*(*i*, *n*) in Equation (22).

When *i* = *c* and *n* = *t*, *J*(*i*, *n*) then is identical to the actual cost function, *J*, given in Equation (7). Therefore, the output of the circuit becomes *J* as the cost function computations for all the training vectors are completed.

### 4.11. FCM Memory Unit of FCM

This unit is used for storing the centers for FCM clustering. There are two memory banks (Memory Bank 1 and Memory Bank 2) in the on-chip centroid memory unit. Memory Bank 1 stores the current centers, **v**_1_, …, **v***_c_*. Memory Bank 2 contains the new centers, **v**_1_, …, **v***_c_*, obtained from the center updating unit. Only the centers stored in the Memory Bank 1 are delivered to the pre-computation unit and membership updating unit for the membership coefficient computation. The updated centroids obtained from the centroid updating unit are stored in Memory Bank 2. Note that the centroids in Memory Bank 2 will not replace the centroids in Memory Bank 1 until all the input feature vectors, **f***_n_*, *n* = 1, …, *t*, are processed.

### 4.12. FCM Operations

**Figure 8.**
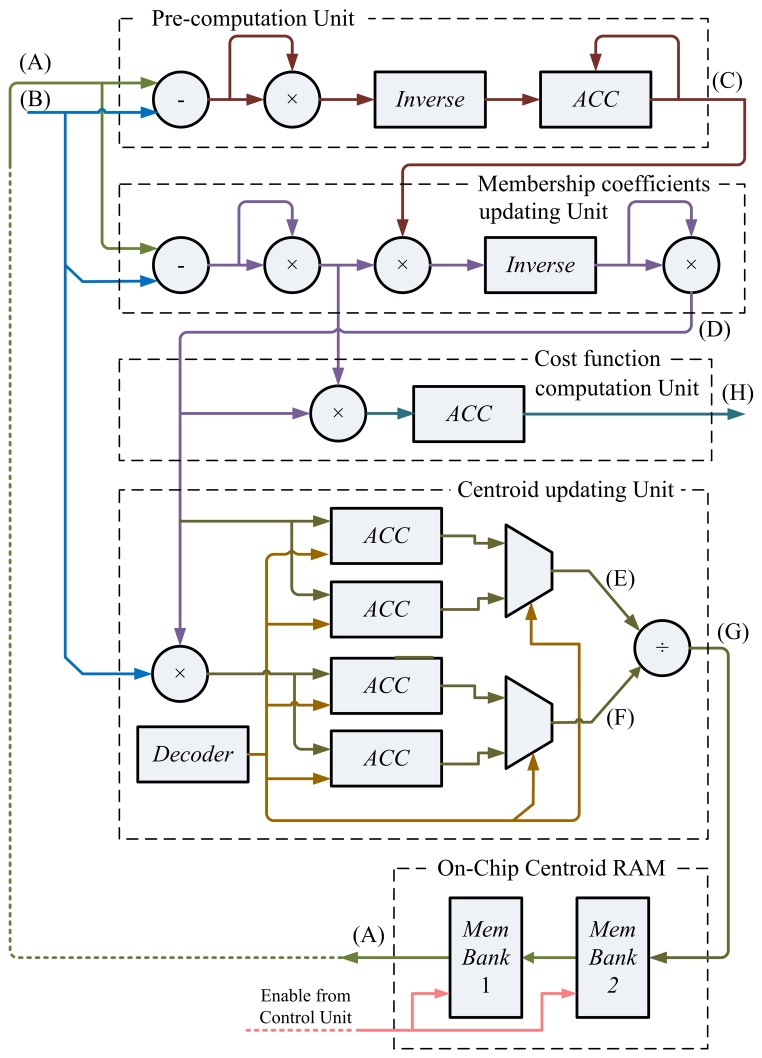
The fuzzy C-means (FCM) circuit for *c* = 2.

For sake of simplicity, Figure 8 shows the FCM circuit for *c* = 2. The circuit therefore is able to separate the set of feature vectors into two classes. The extension of the circuit for larger *c* can be easily accomplished by enlarging the size of the cell array in the center update unit and the memory banks in the memory unit.

The operations of the circuit for *c* = 2 can be separated into four states. Table 1 reveals the operations of each state, which are described in terms of the signals produced at various nodes in the circuit. The locations of the nodes considered in Table 1 are also marked in Figure 8. There are eight nodes (Nodes A–H) under consideration. For sake of brevity, the table considers only the operations of the first two feature vectors (*i.e.*, **f**_1_ and **f**_2_) as examples. Other feature vectors, **f***_n_*, also operate in the same fashion.

In the FCM circuit, each feature vector, **f***_n_*, is retrieved from the GHA circuit. It stays in Node B of the FCM circuit for all four states. Each center, **v***_i_*, is obtained from memory unit of the FCM circuit. It appears in Node A of the FCM circuit.

**Figure 9.**
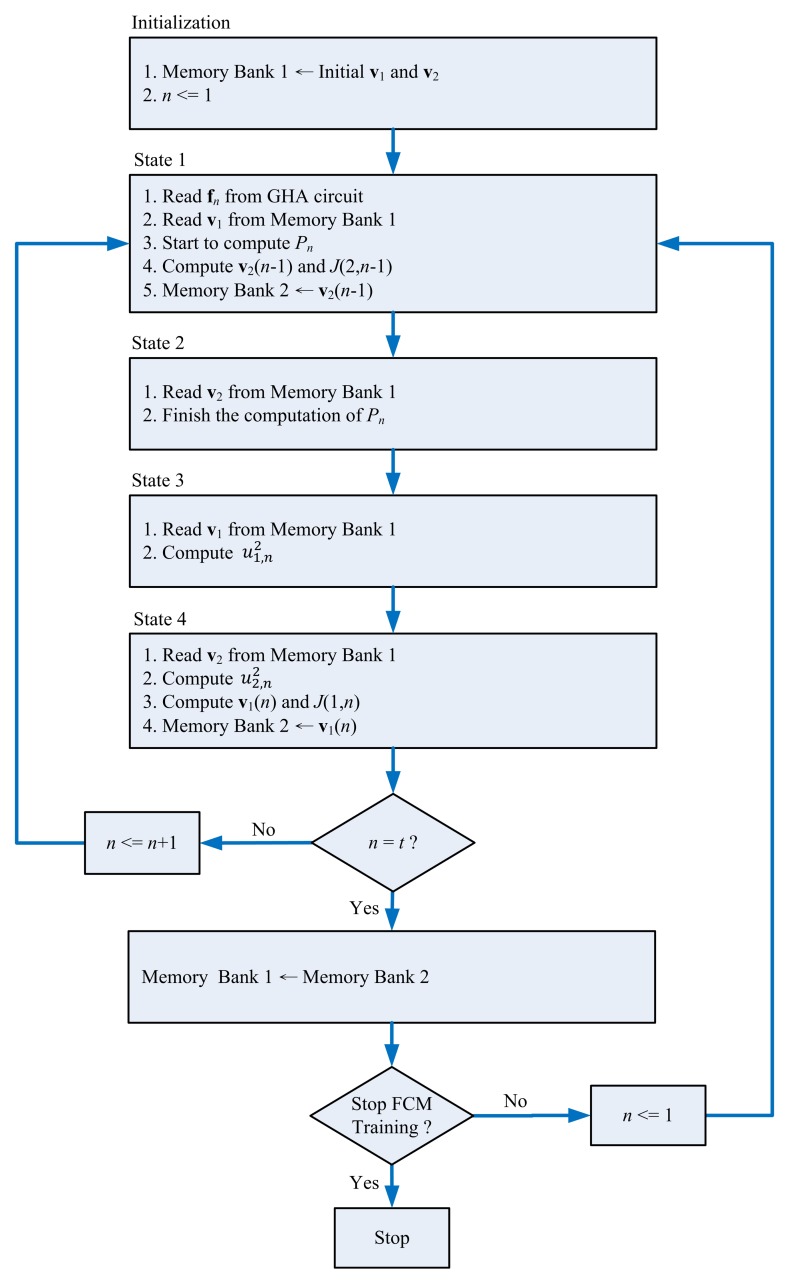
The operations of the FCM circuit for *c* = 2.

Given the feature vector, **f**_1_, it can be observed from Table 1 that the goal of States 1 and 2 is to compute *P*_1_ (shown in Node C) using the precomputation unit. After that, States 3 and 4 compute the membership coefficients, $u_{1,1}^{2}$ and $u_{2,1}^{2}$, using the membership coefficients updating unit, respectively. The membership coefficients are produced in Node D in Figure 8. In addition, based on $u_{1,1}^{2}$ obtained from State 3, the incremental center, **v**_1_(1), and incremental cost, *J*(1, 1), are computed by the center updating unit and cost function computation unit in State 4, respectively. The **v**_1_(1) and *J*(1, 1) are produced at Nodes G and H, respectively. Based on $u_{2,1}^{2}$ obtained from State 4, the **v**_2_(1) and *J*(2, 1) are then computed in State 1 associated with **f**_2_, respectively. The *P*_2_ is also computed in this state. The States 2–4 associated with **f**_2_ then operate in the same manner as those associated with **f**_1_. Figure 9 shows the corresponding state diagram in the controller of the FCM circuit.

### 4.13. Cluster Validity Index Computation

The proposed circuit can be shared by FCM operations with different numbers of clusters (*i.e.*, different *c*'s). This can easily be accomplished by designing the cell array in the center updating unit and the memory bank in the memory unit in accordance with the largest *c*. It is not necessary to modify other parts of the FCM circuit. An advantage of allowing different *c*'s to share the same circuit is that it helps to perform automatic detection of cluster numbers when prior knowledge of the number of clusters is not available.

The proposed method for automatic detection of number of clusters is based on a cluster validity index [24,25]. In this approach, FCM-based clusterings with different numbers of clusters are executed independently. The value of the cluster validity index is computed after each clustering. The selected number of clusters for spike sorting is the number optimizing the cluster validity index.

A number of cluster validity indices have been proposed for FCM. The index used by the proposed circuit is based on a modified partition coefficient. For a given *c*, the partition coefficient, denoted as *I*(*c*), is defined as:

(23) $$I(c)=\sum_{i=1}^{c}\sum_{n=1}^{t}u_{i,n}^{2}+c\times \delta$$

The *c* maximizing *I*(*c*) is then selected as the cluster number for spike sorting. When *I*(*c*) is biased for smaller *c* values (or larger *c* values), a simple compensation function, *c*×*δ*, can be included in *I*(*c*), where *δ* is a constant. In the proposed circuit, the cell array in the centroid computation unit accumulates $u_{i,n}^{2}$. Therefore, the proposed circuit can be used for the computation of *I*(*c*) with only minor modifications. Other cluster validity indices [25] can also be used for the proposed FCM circuit. A dedicated unit for the computation of these indices may be required.

### 4.14. Interaction Between GHA and FCM Circuits

**Figure 10.**
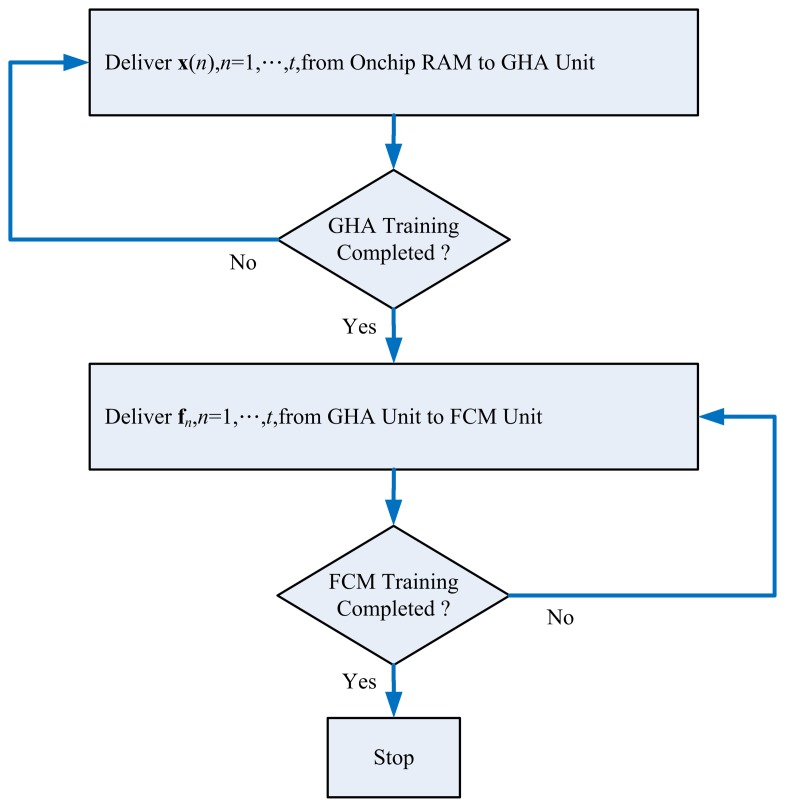
The state diagram of the global controller.

The global controller of the proposed spike sorting circuit shown in Figure 1 is responsible for the interaction between GHA and FCM circuits. Figure 10 shows the state diagram of the global controller. From Figure 10, it follows that the GHA unit will operate first for the feature extraction. The spikes, **x**(*n*),*n* = 1, …,*t*, are delivered to the GHA circuit one at a time for the training of synaptic weight vectors, **w**_1_,..,*p*. The delivery of the same set of spikes, **x**(*n*),*n* =1,…,*t*, will not be halted until the GHA training is completed. Upon the completion of GHA training, the spike set, **x**(*n*), *n* = 1, …, *t*, is delivered to the GHA circuit again for the computation of feature vectors **f***_n_*, *n* = 1, …, *t*.

The FCM circuit will not be activated until all the feature vectors, **f***_n_*, *n* = 1, …, *t*, are available in Buffer D of the GHA unit. After that, the feature vectors, **f***_n_*, *n* = 1, …, *t*, are delivered one at a time to the FCM unit from the GHA unit for the training of centers **v**_1_, …,**v***_c_*. The delivery of the same set of feature vectors, **f***_n_*, *n* = 1, …, *t*, will not be halted until the FCM training is completed. The resulting centers, **v**_1_, …, **v***_c_*, computed by the FCM unit can then be used for the classification of spikes.

### 4.15. SOC-Based Spike Sorting System

**Figure 11.**
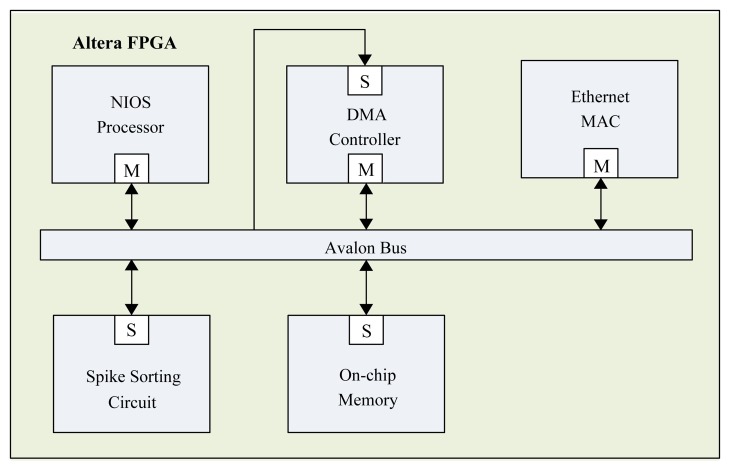
The System-on-Chip (SOC)-based spike sorting system.

**Figure 12.**
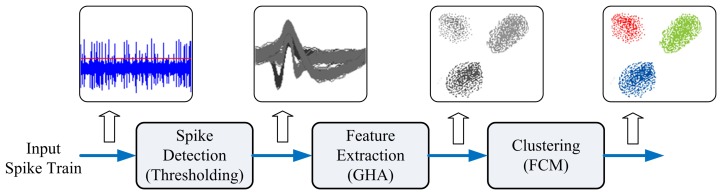
The operations of the SOC-based spike sorting system.

The proposed architecture is used as a custom user logic in an SOC system consisting of a softcore NIOSCPU [26], DMAcontroller, Ethernet MACcontroller and on-chip RAM, as depicted in Figure 11. In a typical spike sorting system, there are three operations: spike detection, feature extraction and clustering; as shown in Figure 12. The nonlinear energy operations (NEO) and thresholding can be used for the spike detection, which is implemented by software running on a NIOS softcore CPU. The operations supported by the proposed architecture are feature extraction and clustering. All the detected spikes are stored in the on-chip RAM and then transported to the proposed spike sorting circuit for both feature extraction and clustering. The DMA-based training data delivery is performed so that the memory access overhead can be minimized. The softcore NIOS CPU coordinates different components in the SOC. It is responsible for circuit activation and control. The results of feature extraction and clustering are stored in the memory unit of the GHA and FCM circuits for subsequent operations.

The Ethernet MAC controller in the proposed SOC-based spike sorting system is responsible for external communication. The inputs are fed to the system via the Internet with a maximum bandwidth of 100 M bits per second at the physical layer. The inputs are spike trains, where 12 bits are used to represent each spike sample. The outputs are the cluster index and/or location of each detected spike.

## 5. Performance Analysis and Experimental Results

In order to evaluate the performance of the proposed architecture for spike sorting, the simulator developed in [23] is adopted to generate extracellular recordings. The simulation gives access to ground truth about spiking activity in the recording and, thereby, facilitates a quantitative assessment of architecture performance, since the features of the spike trains are known *a priori*. Various sets of spikes with different signal-to-noise (SNR) ratios and interference levels have been created by the simulator for our experiments. All the spikes are recorded with a sampling rate of 24,000 samples/s. The length of each spike is 2.67 ms. Therefore, each spike has 64 samples. The dimension of vectors for GHA training, therefore, is *m* = 64. The number of PCs is *p* = 2 for the circuit design.

We first consider the classification correct rate (CCR) of the proposed architecture. The CCR for spike sorting is defined as the number of spikes that are correctly classified by the total number of spikes. To show the robustness of the proposed architecture against noise interference, various SNR ratios are considered, ranging from SNR = 1 dB to 10 dB. Table 2 shows the resulting CCRs for the spike trains with two target neurons (*c* = 2) and three target neurons (*c* = 3). The number of interfering neurons is two. The duration of the spike trains is 14 seconds. The spikes extracted from the spike trains are used for GHA and FCM training, as well as spike classification. The total number of spikes used for training and classification (*t*) and the number of spikes that are correctly classified (*t̄*) are also included in the table. Because the performance of FCM training may be dependent on the selection of initial vectors, each CCR value in the table is the average CCR values of 40 independent executions. From the table, it can be observed that the proposed architecture is able to attains CCR above 84 % for *c* = 3 when SNR = 1 dB.

**Figure 13.**
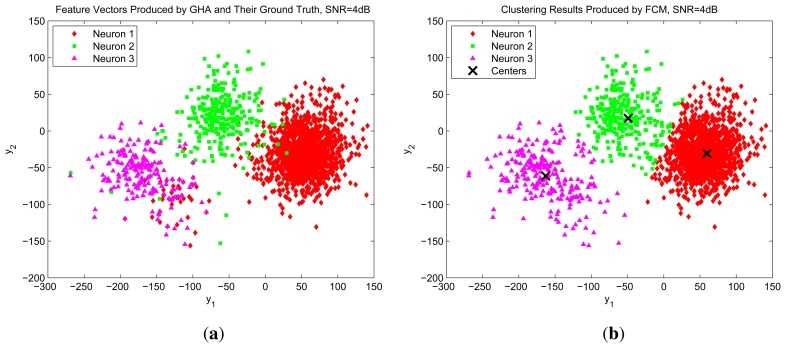
The distribution of GHA feature vectors of spikes, and the results of FCM clustering for SNR = 10. (a) Ground truth of neuron spikes; (b) clustering results produced by FCM.

To further elaborate the effectiveness of the GHA and FCM algorithms for spike sorting, Figure 13 shows the distribution of GHA feature vectors of spikes for SNR = 4 and the results of FCM clustering. The center of each cluster produced by FCM is also marked in the figure. By comparing Figure 13a with Figure 13b, we see that the proposed GHA and FCM circuits are able to correctly separate spikes, even for large noises.

We also use the the distribution of GHA feature vectors in Figure 13a as an example to evaluate the effectiveness of the automatic detection of the number of clusters. The cluster validity index *I*(*c*) defined in Equation (23) is computed for c = 2, 3 and 4, based on the distribution. The computed values, *I*(2), *I*(3) and *I*(4), are 1,594,1,12 and 1,467, respectively. Because *I*(3) has the largest, the estimated cluster number is *c* = 3, which is consistent with the ground truth.

In addition to SNR values, the number of interfering neurons may also influence the CCRs for spike sorting. Table 3 shows the CCRs of the GHA and FCM algorithms for different numbers of interfering neurons *e*. In the experiments, the number of target neurons *c* = 3. It can be observed from Table 3 that only a small degradation is introduced when the number of interfering neurons increases.

The performance of the proposed architecture with imperfect spike detectors is considered in the experiments shown in Table 4 for three target neurons and two interfering neurons. The training sets are obtained from NEO detectors. Therefore, the training sets contain both true positive spikes (*i.e.*, actual spikes) and false positive spikes (*i.e.*, although detected by the detectors, they are actually not spikes). The training results are then applied to the test sets (different from the training set) for CCR measurements. The false positive ratio (FPR) in Table 4 is defined as the number of false positive spikes divided by the total number of spikes in the training set. It can be observed from Table 4 that the CCRs are robust against the FPRs. When the FPRs are below 50 %, the proposed architecture is able to maintain CCRs above 80% for SNR at 1 dB.

In addition to having high CCR, the proposed spike sorting circuit features low area complexities. Table 5 shows the area complexities of the proposed circuit. Because adders, multipliers and registers are the basic building blocks of the GHA and FCM architectures, the area complexities considered in the table are separated into three categories: the number of adders, the number of multipliers and the number of registers. We can see from the table that the number of adders and multipliers grows linearly with the number of PCs, *p*, and number of elements in a block, *q*. They are not dependent on the vector dimension, *m*, the number of clusters, *c*, and the number of training vectors, *t*.

Because the spike sorting circuit contains buffers for storing spikes, synaptic weight vectors, and feature vectors, the number of registers increases linearly with *p*, *m*, *c* and *t*. Table 6 shows the hardware resource utilization for spike sorting applications with *p* = 2, *m* = 64, *c* = 3, and *t* = 800. The design platform for the experiments is the Altera Quartus II with Qsys [27]. The target FPGA device is an Altera Cyclone IV EP4CGX150DF31C7. Three different area resources are considered in the Table 6: logic elements (LEs), embedded memory bits and embedded multipliers. The LEs are used for the implementation of adders, multipliers and registers in the GHA and FCM architectures. The LEs, embedded memory bits and memory bits are used for the implementation of the NIOS CPU of the SOC system. The embedded multipliers are used for the implementation of the multipliers of the GHA and FCM architectures. There are 149,760 LEs, 720 embedded multipliers and 6,635,520 memory bits in the target FPGA device. It can be observed from Table 6 that only limited hardware resources are consumed by the proposed circuit. The NIOS CPU also utilizes hardware resources. However, the hardware costs of the SOC are only slightly larger than those of the spike sorting circuit, as shown in Table 6.

The proposed SOC-based spike sorting system features fast computation. Table 7 shows the training time of the proposed spike sorting system for various clock rates. The training time of its software counterpart running on the Intel I7 processor is also included in the table for comparison purposes. The training set for these experiments consists of 800 spikes. The number of epochs for GHA training is 100. The number of iterations for FCM training is 10. It can be observed from Table 7 that the propose SOC-based spike sorting system is able to operate up to a 1 GHz clock rate. In addition, both the GHA and FCM training times decrease linearly with the clock rate. When clock rate becomes 1 GHz, the total training time of the proposed SOC-based spike sorting system is only 1.99 ms. By contrast, the training time of the Intel I7 processor is 193.18 ms. The speedup of the proposed hardware system over its software counterpart is therefore 97.08.

To further facilitate the spike sorting computation, the computation of cluster validity index is also implemented by hardware. The cluster validity index given in Equation (23) mainly contains the sum of membership coefficients. Because the FCM circuit is able to provide the sum of membership coefficients for each cluster without additional overhead, the major operations of the cluster validity index circuit are only to the collect cluster validity indices for different number of clusters. After all the indices are available, the circuit then finds the index with the largest value. As a result, the latency of the circuit for computing *I*(*c*) is only four clock cycles (*i.e.*, 4 ns at 1 GHz). The circuit also consumes only 227 logic elements. No embedded multiplier and memory bit are required. The proposed cluster validity index is therefore beneficial for enhancing the ability to automatically select the right number of clusters at the expense of low latency and area overhead.

The proposed architecture can be directly used for offline data analysis, where the stored raw spike trains can be used as the inputs. The proposed architecture then performs fast GHA and FCM training for real-time spike sorting with training time 1.99 ms at a 1 GHz clock rate. For the software-based spike sorting systems, such as Wave Clus [28], the total computation time (measured from the Intel I7 2.61 GHz processor) for feature extraction and clustering is 464.35 ms for the same dataset. Direct comparisons between the proposed architecture and Wave Clus may be difficult, because they are based on different algorithms for spike sorting. However, the superiority in the computation speed of the proposed system can still be observed. From Table 7, we see that the proposed architecture has longer computation time, only when it operates at a slower clock rate of 1 MHz.

The power consumption of the proposed architecture implemented by FPGA at different clock rates is included in Table 8. The power consumption estimation is based on the tool, PowerPlay Power Analyzer, provided by Altera. It can be observed from Table 8 that the power consumption is 91.75 mW when the clock rate is 1 MHz. Therefore, the architecture at 1 MHz may be suited for online applications with limited power resources. The power consumption is 5.84 W when the architecture operates at 1 GHz. An advantage of the proposed architecture operating at a higher clock rate is that it is able to perform GHA and FCM training for a larger number of channels per unit time. To show this fact, the number of channels the proposed architecture can process per second for each clock rate is also included in Table 8. It can be observed from the table that the number of channels per second is 502.5 for a clock rate of 1 GHz. In this case, the proposed architecture is suited for real-time offline operations.

We have also implemented the architecture by ASIC as implantable chips. The implementation is based on Sypnosys Design Compiler with TSMC90 nm technology library. The power estimation is based on PrimeTime provided by Sypnosys. The estimated power dissipation of the ASIC is only 440.34 μW at a 1 MHz clock rate, which is significantly less than the power consumption of its FPGA counterpart (*i.e.*, 91.75 mW) at the same clock rate. The total area of the proposed architecture is 0.7432 mm^2^. The power density, therefore, is 59.25 mW/cm^2^. It is recommended that the power density should be below 80 mW/cm^2^ [29], so that potential brain damage can be avoided. The ASIC version of the proposed spike sorting system has a power density below 80 mW/cm^2^; therefore, it may be well suited as an implantable chip at the front end.

Next, we compare the proposed GHA and FCM circuits with existing implementations. Table 9 compares the average CCRs of the various algorithms adopted by [9,10,12] for different SNR ratios. All the algorithms are evaluated with the same set of parameters (*i.e.*, *c* = 3, *p* = 2, *m* = 64). Given an SNR value, the CCRs for different algorithms are measured on the same noisy spike trains. To obtain the average CCR, the algorithms are executed 40 times for each SNR value, independently. It can be observed from Table 9 that the combination of GHA and FCM for spike sorting outperforms other algorithms. As compared with the K-Means algorithm, the FCM algorithm is less sensitive to the selection of initial centers for clustering. Therefore, the FCM-based spike sorting algorithms have higher CCRs, as revealed in the table. This is especially true when SNR values are high (e.g., 10 dB). In fact, when noise energy becomes lower, the feature vectors produced by GHA or PCA algorithms are located in smaller regions. As a result, it is more likely that some of the randomly selected initial centers are far away from these regions. The K-means algorithm is then likely to fall into a poor local optimum. Given the same algorithm for clustering (such as FCM), the GHA attains comparable performance with PCA. Therefore, the combination of GHA with FCM is an effective alternative for spike sorting.

In Table 10, we compare the area costs and throughput of the proposed GHA circuit with those of other FPGA-based hardware implementations [12,30] for feature extraction. The throughput is defined as the number of input training vectors the circuit can process per second. It can be observed from Table 10 that the proposed GHA architecture attains the highest clock rate and throughput. In fact, the proposed architecture has a throughput 28.125 (4.50 × 10^7^ *vs.* 1.60 × 10^6^) and 16.3 times ((4.50 × 10^7^ *vs.* 2.75 × 10^6^) higher than that of the architectures in [12,30], respectively. The proposed algorithm has superior performance, because it is based on shift registers for storing weight vectors and input vectors for high-speed computation.

Finally, Table 11 reveals the area costs and throughput of various clustering implementations. The throughput is also defined as the number of input training vectors the FCM architecture can process per second. The proposed architecture consumes more hardware resources. However, the FCM architecture in [20] is implemented only for two clusters (*c* = 2). Therefore, the architecture is applicable only to two target neurons. By contrast, the proposed circuit is implemented for three clusters (*c* = 3). Therefore, with throughput 10.3 times (3.38 × 10^7^ *vs.* 3.28 × 10^6^) higher, the proposed architecture is also able to perform FCM with a larger number of clusters as compared with the work in [20]. All these facts demonstrate the effectiveness of the proposed hardware spike sorting system.

## 6. Concluding Remarks

The proposed architecture has been implemented on the Altera FPGA Cyclone IV for physical performance measurement. The architecture is used as an hardware accelerator to the NIOS CPU in an SOC platform. Experimental results reveal that the proposed spike sorting architecture has the advantages of high CCR and high computation speed. For SNR = 10, its CCR is above 96% for three target neurons. When SNR becomes 1 dB, it is still able to retain CCR above 84%. The architecture is able to achieve a 1 GHz clock rate. The speedup over its software counterpart running on the Intel I7 processor is above 97. Its GHA and FCM circuits have a higher computation speed compared with existing hardware implementations for feature extraction and clustering. In particular, the proposed GHA circuit has throughput 16.3 times higher than that of the existing implementations. These results show that the proposed system implemented by FPGA is an effective real-time training device for spike sorting.

## Figures and Tables

**Table 1.** Operations of the FCM unit for the first two feature vectors, f_1_ and f_2_.

**State**	**Node Name**							
	**A**	**B**	**C**	**D**	**E**	**F**	**G**	**H**
State 1	**v**_1_	**f**_1_	×	×	×	×	×	×
State 2	**v**_2_	**f**_1_	*P*_1_	×	×	×	×	×
State 3	**v**_1_	**f**_1_	*P*_1_	$u_{1,1}^{2}$	×	×	×	×
State 4	**v**_2_	**f**_1_	*P*_1_	$u_{2,1}^{2}$	$u_{1,1}^{2}$	$u_{1,1}^{2}{\text{f}}_{1}$	**v**_1_(1)	*J*(1, 1)
State 1	**v**_1_	**f**_2_	×	×	$u_{2,1}^{2}$	$u_{2,1}^{2}{\text{f}}_{1}$	**v**_2_(1)	*J*(2, 1)
State 2	**v**_2_	**f**_2_	*P*_2_	×	×	×	×	*J*(2, 1)
State 3	**v**_1_	**f**_2_	*P*_2_	$u_{1,2}^{2}$	×	×	×	*J*(2, 1)
State 4	**v**_2_	**f**_2_	*P*_2_	$u_{2,2}^{2}$	$\sum_{k=1}^{2}u_{1,k}^{2}$	$\sum_{k=1}^{2}u_{1,k}^{2}{\text{f}}_{k}$	**v**_1_(2)	*J*(1, 2)

**Table 2.** Classification correct rates (CCRs) of the proposed architecture for the spike sorting with different signal-to-noise (SNR) levels.

		**SNR (dB)**					

		**1**	**2**	**4**	**6**	**8**	**10**
*c*=2	*t*	1,651	1,638	1,621	1,656	1,662	1,653
	*t̄*	1,644	1,632	1,617	1,654	1,660	1,651
	CCR	99.58%	99.63%	99.75%	99.88%	99.88%	99.88%
*c*=3	*t*	1,850	1,860	1,842	1,870	1,873	1,828
	*t̄*	1,571	1,672	1,737	1,791	1,812	1,769
	CCR	84.92%	89.89%	94.30%	95.78%	96.74%	96.77%

**Table 3.** The CCRs of the GHA and FCM algorithms for different number interfering neurons.

**SNR (dB)**	**1**	**2**	**4**	**6**	**8**	**10**
*e* = 2	84.92%	89.89%	94.30%	95.78%	96.74%	96.77%
*e* = 7	83.24%	87.74%	92.91%	94.58%	95.50%	95.82%

**Table 4.** The CCRs of the GHA and FCM algorithms for spike detectors with different FPRs at SNR = 1 dB.

FPR	29.86%	34.51%	41.30%	45.46%	53.66%

CCR	83.48%	82.17%	81.18%	80.58%	78.34%

**Table 5.** Area complexities of the proposed spike sorting circuit.

	**GHA**	**FCM**	**Total**
Adders	*O*(*q*)	*O*(*p*)	*O*(*p* + *q*)
Multipliers	*O*(*q*)	*O*(*p*)	*O*(*p* + *q*)
Registers	*O*(*pm* + *pt*)	*O*(*pc*)	*O*(*p*(*m* + *t* + *c*))

**Table 6.** Hardware resource utilization of the proposed spike sorting systems.

	**GHA** **Circuit**	**FCM** **Circuit**	**Spike Sorting** **Circuit**	**Entire** **SOC**
Logic	15,688	4,468	22,582	31,018
Elements	(10.48%)	(2.98%)	(15.08%)	(20.71%)
Embedded	128	23	151	155
Multiplier	(17.78%)	(3.19%)	(20.97%)	(21.53%)
Memory	63,488	113,520	193,444	1,050,380
Bits	(0.96%)	(1.71%)	(2.91%)	(15.83%)

**Table 7.** The training time of the proposed spike sorting system for various clock rates.

**Implementation**			**SOC- Based** **Spike Sorting**				**Software** **Spike Sorting**
Processor			Altera NIOS II				Intel I7
Clock Rate	1 MHz	10 MHz	50 MHz	200 MHz	800 MHz	1 GHz	2.61 GHz

GHA (ms)	1,776.51	177.81	35.60	8.92	2.23	1.78	181.38
FCM (ms)	204.33	20.60	4.17	1.05	0.26	0.21	11.80
Total (ms)	1,980.84	198.4	39.77	9.97	2.49	1.99	193.18

**Table 8.** The estimated power consumption of the proposed GHA and FCM circuits implemented by field programmable gate array (FPGA) at different clock rates.

**Clock Rate**	**1 MHz**	**10 MHz**	**50 MHz**	**200 MHz**	**400 MHz**	**800 MHz**	**1 GHz**
Est.Power (mW)	91.75	106.71	179.06	429.36	805.70	2,985.00	5,834.34
Channels per Second	0.5	5.0	25.1	100.3	201.1	401.6	502.5

**Table 9.** CCR values of various spike sorting algorithms. PCA, principal component analysis.

	**Proposed** **GHA+FCM**	**GHA +** **K-Means** [12]	**PCA +** **FCM** [9]	**PCA +** **K-Means** [10]
SNR = 1 dB	84.92%	85.23%	84.21%	85.10%
SNR = 4 dB	94.30%	93.13%	94.08%	92.66%
SNR = 6 dB	95.78%	94.65%	95.72%	93.42%
SNR = 8 dB	96.74%	96.74%	96.69%	96.74%
SNR = 10 dB	96.77%	86.93%	96.72%	89.67%

**Table 10.** Comparisons of the proposed GHA circuit with other FPGA-based feature extraction implementations. LE, logic element.

**Arch.**	**FPGA** **Devices**	**Logic Cells** **or LEs**	**DSPelements** **or Multipliers**	**Embedded** **Bits**	**Maximum Clock** **Rate**	**Throughput**
Proposed GHA Arch.	Altera Cyclone IV EP4CGX150	15,688	128	63,488	1 GHz	4.50 × 10^7^
GHA Arch. in [12]	Xilinx Virtex 6 XC6VSX315T	12,610	12	0	100 MHz	1.60 × 10^6^
GHA Arch. in [30]	Xilinx Cyclone IV EP4CGX150	9,144	432	63,448	50 MHz	2.75 × 10^6^

**Table 11.** Comparisons of the proposed FCM circuit with other FPGA-based clustering implementations.

**Arch.**	**FPGA Devices**	**Logic Cells or LEs**	**DSP elements or Multipliers**	**Embedded Bits**	**Maximum Clock Rate**	**Throughput**
Proposed FCM Arch.	Altera Cyclone IV EP4CGX150	4,468	23	113,520	1 GHz	3.38 × 10^7^
FCM Arch. in [20]	Altera ACEX 1K EP1K100FC484	4,205	0	24,576	NA	3.28 ×10^6^
